# A Low-Cost Process for Fabricating Reinforced 3D Printing Thermoplastic Filaments

**DOI:** 10.3390/polym15020315

**Published:** 2023-01-07

**Authors:** Mohamed Hassanien, Maen Alkhader, Bassam A. Abu-Nabah, Wael Abuzaid

**Affiliations:** Department of Mechanical Engineering, American University of Sharjah, Sharjah P.O. Box 26666, United Arab Emirates

**Keywords:** 3D printing, reinforced filaments, FDM, filament extrusion, composite filaments

## Abstract

Low-cost desktop-sized fused deposition modeling (FDM) printers have been widely embraced by small to large-scale institutions and individuals. To further enhance their utility and increase the range of materials that they can process, this work proposes a low-cost solution that adapts to low-cost desktop-sized extruders and enables them to fabricate filaments comprising a wide range of nonorganic reinforcing particles. This solution will fill a gap in the field, as low-cost fabrication techniques for reinforced filaments have been lacking. In the proposed solution, particles are heated and deposited on thermoplastic pellets to form a coating. Coated pellets are subsequently extruded using a low-cost desktop single-screw extruder. The effectiveness of the process is demonstrated by fabricating polylactic acid (PLA) filaments reinforced with two types of reinforcements, namely, dune sand and silicon carbide. Filaments’ stiffness and strength were measured, and their microstructure along their lateral and longitudinal directions were investigated. Improvements in tensile strength (up to 8%) and stiffness (up to 4.5%) were observed, but at low reinforcement levels (less than 2 wt%). Results showed that the proposed process could be used to fabricate filaments with multiple types of particles. The produced filaments were successfully used to fabricate 3D parts using a commercial desktop FDM printer.

## 1. Introduction

Additive manufacturing techniques have become ubiquitous in the engineering and industrial scenes. The growing dependence on them stems from their proven utility in fabricating components with very complex 3D geometries and from a wide range of materials [1,2,3,4,5,6]. Moreover, they eliminate the need for subtractive processes or molds, which allows them, particularly in nonmass production cases involving complex shapes, to deliver a simpler, faster, less expensive, and more sustainable manufacturing solution [7]. 

Each of the additive manufacturing techniques available today has its own unique advantages that make it favored in certain applications [3]. However, in terms of the number of users and community size, the most used additive manufacturing technique is fused deposition modeling (FDM), also known as fused filament fabrication (FFF) [7]. In FDM printers, a filament is heated in the printer head and layers of it are deposited on a printing platform. This process is repeated to build the designed 3D part layer by layer [7]. The described FDM printing process is inherently simple and can be realized at a low cost. Thus, it has been used in developing small-scale, low-cost 3D printers (i.e., desktop 3D printers). These 3D printers have brought about a paradigm shift in the role and impact of FDM 3D printing technology. Desktop FDM 3D printers provide an accessible, low-cost enabling platform to visualize and improve designs, fabricate custom parts, enhance creativity, and accelerate innovation. Accordingly, they have been widely embraced by scientists, engineers, academics, students, designers, and enthusiasts. Currently, desktop FDM 3D printers often serve as the gateway through which users enter the world of 3D printing and additive manufacturing. 

Desktop FDM 3D printers are compatible with multiple commercially available polymeric filaments. The most common are polylactic acid (PLA) and acrylonitrile butadiene styrene (ABS). The list of commercially available filaments is continuously expanding to satisfy the needs of the desktop FDM 3D printing community. However, newer types of filaments are moving toward including different phases and reinforcements rather than using a monolithic polymeric constituent. Multiphase filaments can be designed to provide application-tailored properties such as enhanced stiffness, strength, ductility, thermal and electrical conductivities, dielectric behavior, and piezoelectric properties. These enhanced properties can accelerate the development of 3D-printed lightweight structures, sensors and actuators [8,9,10,11,12,13]. 

Examples demonstrating the progress in multiphase filaments are abundant in the literature. PLA filaments reinforced with talc particles were developed to realize 3D-printed parts with higher stiffness [14]. Including 3% talc in PLA filaments increased PLA’s flexural modulus by 14.7%. PLA filaments comprising MgAl_2_O_4_:Sm^3+^ were developed to form phosphor bioplastics that can produce parts with luminescent properties [15]. Cork-PLA composite filaments were developed to provide enhanced impact resistance properties [16]. Carbon nanotubes (CNT) were incorporated into PLA filaments to enhance their tensile strength, elongation at break, impact strength, and thermal stability [17]. PLA filaments comprising different weight fractions of short carbon fibers were developed to provide enhanced fracture toughness behavior [18]. Micro- and nanocellulose fibers were incorporated into PLA composite filaments to produce filaments with improved mechanical, biodegradability, and sustainability characteristics [19]. 

ABS filaments reinforced with different phases were also developed. Polycarbonate (PC) and graphene pellets [20] as well as multiwall carbon nanotubes (MWCNT) [21] were incorporated into ABS filaments to increase their stiffness, strength, transition temperature, and electrical conductivity. ABS filaments reinforced with long carbon fibers were manufactured to provide substantially improved mechanical and thermal properties [22]. ABS filaments comprising iron and copper were developed to deliver enhanced thermomechanical properties such as increased thermal conductivity and reduced thermal capacity [23]. ABS filaments with thermoelectric properties and improved dielectric properties were fabricated by incorporating Bi_2_Te_3_ [24] and BaTiO_3_ [25,26], respectively. 

The properties of nylon and polyethylene (PE) filaments were also modified by adding reinforcement. Nylon filaments were reinforced with Al_2_O_3_ [27] and short fibers (i.e., Kevlar, carbon, and glass) [28] to improve their stiffness, strength, and fatigue life. PE filaments were reinforced with nickel and tin particles [29], alumina whiskers [30], and fly ash [31] to increase their electrical conductivity, thermal conductivity, and stiffness-to-weight ratio, respectively. 

The aforementioned examples, which are not exhaustive of all types of reinforced FDM-compatible filaments, demonstrate the growing demand for reinforced thermoplastic filaments. However, fabricating reinforced thermoplastic filaments is complex, as it typically requires multistage extruders comprising twin extruding screws [14,15,16,21,32,33]. The twin-screw configuration is required to thoroughly mix the reinforcing elements with the plastic pellets (e.g., PLA or ABS) prior to extruding them into filaments. Such extruders are industry and large institution-oriented, expensive, and of large size; therefore, they are inaccessible to a large segment of the desktop FDM 3D printers’ community. This motivated this work to propose an alternative, low-cost process for fabricating reinforced filaments. The proposed process aims to provide users of desktop FDM 3D printers with an accessible, low-cost solution that allows them to develop in-house customized reinforced filaments. 

**Figure 1:**
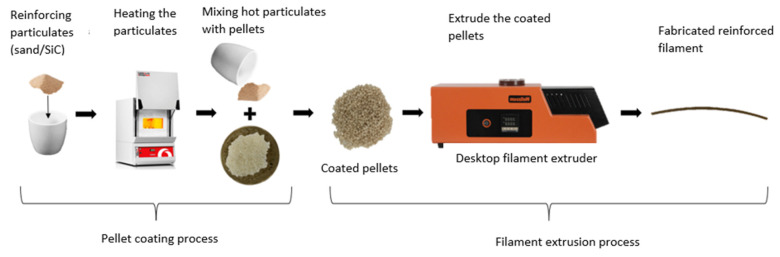
Schematic diagram describing the proposed premixing and extrusion processes.

The proposed process, schematically described in Figure 1, utilizes low-cost desktop single-screw extruders, which have been used by users of desktop FDM 3D printers to fabricate single-phase plastic filaments from pellets (e.g., the extruders from www.filastruder.com (accessed on 15 October 2022) and www.wellzoomextruder.com (accessed on 15 October 2022). However, existing desktop extruders comprise short nozzles and single extruding screws; accordingly, they are incapable of fabricating a well-mixed multiphase filament. To overcome the mixing problem, this work proposes using pellets premixed with reinforcing particles. The premixed composite pellets consist of pellets coated with the reinforcing particles. To minimize the cost of the process and avoid adding adhesives, which can affect the filament’s properties, pellets are coated by manually mixing them with preheated reinforcing particles. The heated particles adhere to the pellets by locally melting them. The heating step limits the proposed process to inorganic reinforcement that has higher melting temperatures than that of the plastic pellets. The following sections demonstrate its viability through two case studies. In addition, the two case studies were used to investigate the effect of reinforcing particles on the stiffness and strength of polylactic acid (PLA) filaments. 

## 2. Materials and Methods

In this work, a low-cost process for fabricating reinforced filaments compatible with generic desktop FDM 3D printers is proposed. To demonstrate the viability and potential of the proposed process, a proof-of-concept exercise was conducted using two case studies. The methodology used in the proof-of-concept exercise comprises two stages. The first stage demonstrates in detail the proposed fabrication process. This stage shows the practicality and simplicity of the process. The second stage evaluates the quality of the filaments fabricated in the first stage. Filaments’ quality is assessed through their mechanical properties and the reinforcing particle distribution in them. The two case studies used are performed in tandem to minimize redundant efforts. Consequently, the results of the first case study helped in refining the parameters used in the second case study. The following sections describe the employed filament fabrication process as well as the mechanical and optical testing protocols.

### 2.1. Fabrication of Particle Reinforced Filaments Using Single-Screw Extruders 

Two case studies (i.e., two types of reinforced filaments) are used to demonstrate the proposed reinforced filament fabrication process. PLA 4032D, which is a type of polylactic acid, is used to fabricate the thermoplastic filaments for both case studies. PLA is widely used within the 3D printing community due to its low cost, low melting temperatures, compatibility with most commercial FDM printers, and desirable environmentally friendly characteristics (e.g., biodegradability). PLA 4032D is acquired in the form of pellets with a nominal size of 3 mm. The reinforcing particles used in the first and second case studies were dune sand and silicon carbide (SiC), respectively. Dune sand is selected for its abundance and low cost compared to other types of fillers, not for any unique mechanical properties. Therefore, dune sand is used as merely an example material in the proof-of-concept exercise to show that the proposed process can work with a wide range of inorganic reinforcing particles. Dune sand is sourced from the United Arab Emirates desert, which mainly consists of silicates, such as silicon dioxide (SiO_2_), in addition to carbonates and quartz [34]. However, silicon carbide (SiC), which is supplied by McMaster-CARR (Elmhurst, IL, USA), is selected as a reinforcement due to its superior mechanical properties (i.e., high stiffness and strength) [35,36]. The sand and SiC particles used have an average particle size of 150 µm and 53 µm, respectively. 

To fabricate a reinforced filament with a desired reinforcement weight fraction (wt%), reinforcing particles and PLA pellets with the weights of M_R_ and M_P,_ respectively, are used. The filament reinforcement weight fraction (wt%) is calculated as:

(1) $$\mathrm{wt}\%=\frac{M_{R}}{M_{p}+M_{R}}\times 100\%,$$

**Figure 2:**
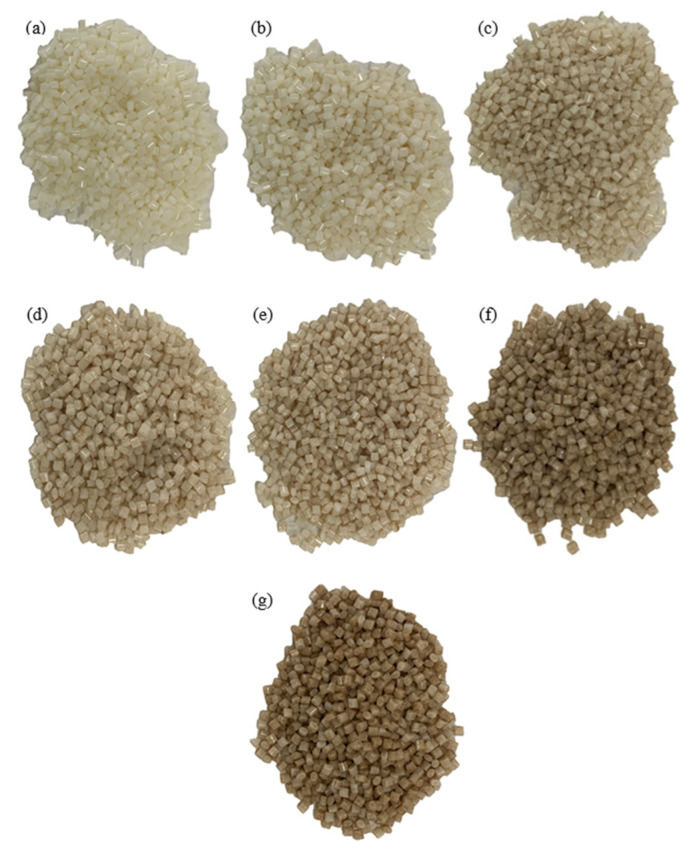
PLA pellets coated with sand particles, showing PLA-sand pellets with sand weight fraction of (**a**) 0 wt%, (**b**) 0.5 wt%, (**c**) 2 wt%, (**d**) 3 wt%, (**e**) 4 wt%, (**f**) 10 wt%, and (**g**) 15 wt%.

**Figure 3:**
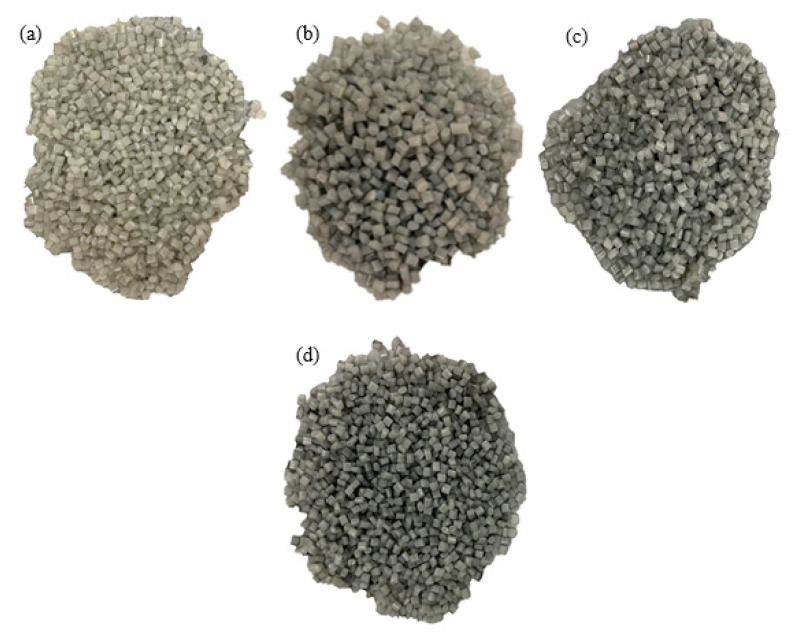
PLA pellets coated with SiC particles, showing PLA-SiC pellets with SiC weight fractions of (**a**) 0.5 wt%, (**b**) 1 wt%, (**c**) 1.5 wt%, and (**d**) 2 wt% SiC.

To satisfy the low-cost objective of this work, a small desktop single-screw filament extruder (Wellzoom, China) is utilized. The extruder has a short nozzle (14 cm) and a single extruding screw; hence, it cannot properly mix the reinforcing particles with the plastic pellets during the extrusion process; the two phases would separate. The used extruder exemplifies the typical desktop extruders used by the desktop FDM 3D printers’ community. As the single-screw extruder does not sufficiently mix the reinforcing particles with the plastic pellets, the pellets and reinforcing particles should be fed to the extruder in a premixed form, as illustrated in Figure 1. To this end, this work proposes to coat the pellets with the reinforcing particles to create a premixed particle-pellets mixture. Subsequently, the coated pellets can be fed to the extruder. Coating the pellets with the reinforcing particles relatively substitutes the mixing process that would take place inside an industrial twin-screw extruder. Coating the pellets requires binding the particles to the pellets. To avoid introducing binding materials (e.g., adhesives), which could affect the behavior of the reinforced filaments, binding the reinforcing particles to the plastic pellets is achieved by locally melting the pellets’ surfaces; that is, following the principles of thermal spray processes. To this end, the reinforcing particles are placed in an electrical oven, and their temperature is raised to 300 °C. The particles are kept in the oven for an hour at the set temperature to ensure they reach uniform and steady-state conditions. Afterwards, the reinforcing particles are gradually poured and manually mixed with the polymeric pellets. Mixing is performed using a crucible and a handheld ceramic stirrer. Before mixing, the pellets are kept at room temperature. During the manual mixing process, the hot reinforcing particles locally melt the pellets and adhere to their surfaces. Manual mixing is continued until all added particles adhere to the pellets, forming composite pellets. To ensure that the desired reinforcement weight percentage is reached, the composite pellets are weighed, and their weight is compared to the weight of the pellets and reinforcing particles used. The aforementioned approach is used to prepare sand-coated PLA pellets with the reinforcement weight fractions of 0.5, 2, 3, 4, 10, and 15 wt% as well as SiC-coated PLA pellets with the reinforcement weight fractions of 0.5, 1, 1.5, and 2 wt%. A maximum SiC reinforcement weight fraction of 2 wt% is used in the second case study as the results of the PLA-sand-reinforced filaments, which are shown later, demonstrating that reinforcement weight fractions exceeding 2 wt% result in unfavorable mechanical properties. Figure 2 and Figure 3 show the sand-coated PLA pellets and SiC-coated PLA pellets, respectively. Darker coated pellets in the figures include higher sand content. The uniform color across each subfigure in Figure 2 and Figure 3 visually demonstrates that the pellets are uniformly coated. Figure 2 includes an image of uncoated PLA pellets, referred to as 0 wt%, to serve as a benchmark. 

Coating PLA pellets with SiC particles involves additional challenges as compared to the cases involving sand. SiC particles are smaller in size and have higher thermal conductivity than sand. Accordingly, heated SiC particles are prone to losing their stored thermal energy rather quickly through convection. Rapid energy loss, which can lead to a significant drop in particles’ temperature during the particle-PLA mixing process, limits the localized melting at the pellets surfaces and leads to poor adhesion between the SiC particles and the pellets. This problem presents itself in the form of leftover SiC particles at the bottom of the crucible after manual mixing concludes. In such a case, the leftover particles are heated again to 300 °C and the mixing process is repeated. Usually, two rounds of mixing were effective in overcoming the quick thermal loss problem faced with SiC particles. 

**Figure 4:**
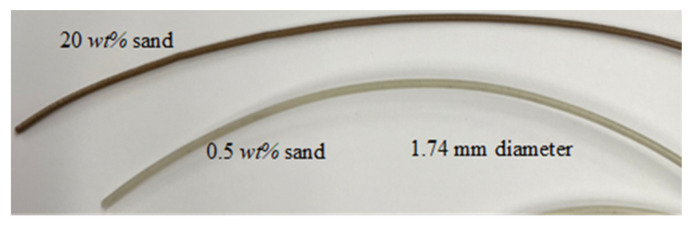
Sample of the fabricated PLA-sand filaments with 20 and 0.5 wt% sand.

Once the coated pellets are prepared, they are placed in the hopper of a Well Zoom Type B single-screw extrusion machine to form 1.75 mm filaments compatible with generic desktop FDM 3D printers. The extrusion temperature and speed are set to 190 °C and 650 mm/min, respectively. The prepared filaments are directly taken for printing and/or testing to avoid prolonged exposure to humidity, which accelerates the biodegradation process of PLA and alters the filaments mechanical properties. The extrusion machine is flushed and purged after each run to prevent contamination. PLA-sand filaments with sand weight fraction of 0, 0.5, 2, 3, 4, 10 and 15 wt% were fabricated first. Subsequently, PLA-SiC filaments with SiC weight fractions of 0, 0.5, 1, 1.5 and 2 wt% were fabricated. Samples of the fabricated reinforced filaments are shown in Figure 4. 

### 2.2. Inspection of Reinforcing Particles Distribution 

**Figure 5:**
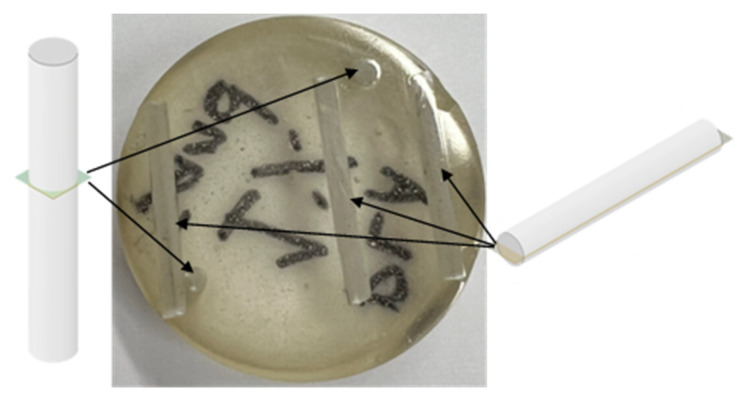
Polished samples for microscopy tests, showing the polished side of longitudinal and vertically aligned samples.

Optical microscopy is used to inspect particle distribution in reinforced filaments fabricated using the proposed process. This step is required to ensure that particles are randomly dispersed in fabricated filaments. Random particle distribution and lack of particle clustering are indications that the reinforcing particles were well-mixed with the PLA pellets during fabrication. The latter, once confirmed, demonstrates that coated-pellets can be used to replace the expensive mixing stage requiring two-stage long-nozzle industrial extruders. Accordingly, optical microscopy images will serve as a tool that provides a visual quality check on the viability of the fabrication process. Particles distribution along the length and width of reinforced filaments is investigated. To this end, vertically and horizontally aligned samples corresponding to filaments with different reinforcement weight fractions are embedded in metallurgical resin molds (i.e., holders), see Figure 5. Subsequently, the molds are polished using an automatic grinding and polishing machine (Metkon Digiprep 251, Bursa, Turkey) using SiC paper with different grit size (P80, 240, 800, 1200, 2400). Polishing is conducted in a wet environment using polishing cloth (i.e., tap water during early polishing stages and colloidal silica suspension during final polishing stages). Polished specimens are inspected using a digital optical microscope (Zeiss Smartproof 5).

### 2.3. Mechanical Characterization of Reinforced Filaments Properties 

**Figure 6:**
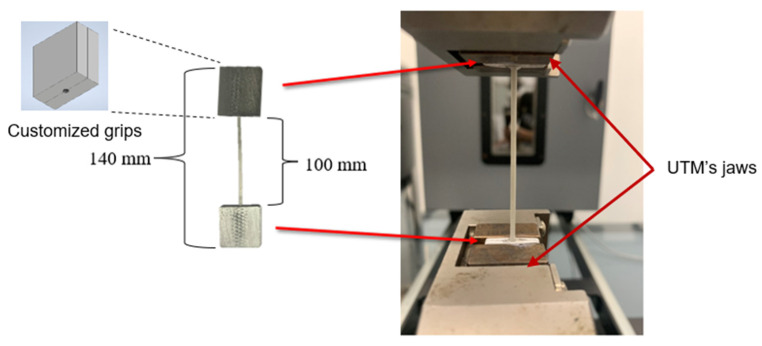
Filament testing using an Instron UTM with custom grips.

To assess the mechanical performance of the developed reinforced filaments, their stress-strain behavior is measured using an Instron Universal Testing Machine (UTM). Since the standard grips of UTM machines can introduce stress localization in the 1.7 mm-wide polymeric filaments, custom grips are designed and fabricated. The custom grips are developed from aluminum and designed to have, when closed, the dimensions of 20 × 20 × 4 mm^3^. They include a 2 mm-diameter groove in the middle. The grips comprise two symmetric sides, as shown in Figure 6. Each sample requires two grips, one at each end. Before placing the filament sample in the grips, superglue is applied to the grips. Superglue assists in preventing slippage during testing. It is used to avoid roughening the surfaces at the filaments’ ends, which is needed to enhance grip. Roughening the surfaces was tried and was associated with localized failure near the grips; thus, it was avoided. The overall and gauge lengths of each sample are 100 and 60 mm, respectively. Filament samples are loaded using a displacement-controlled protocol at a displacement rate of 2 mm/min, which corresponds to a strain rate of approximately 5.6 × 10^−4^ s^−1^. For each reinforced filament, at least four samples are tested. Stress (σ) in tested filaments is computed by dividing the force (F) reported by the Instron’s load cell by the cross-sectional area of the tested filament. On the other hand, strain (ε) in tested filaments is computed by dividing the crosshead displacement (u) reported by the Instron’s actuator by the gauge length of the tested specimen. The crosshead displacement accurately represents the deformation in the samples, as the machine stiffness is orders of magnitude higher than that of the tested samples. The maximum load used in testing the samples is less than 2% of the machine’s capacity. At such loads, the testing machine’s compliance can be ignored. The filaments’ diameter slightly varied along their length (±0.02 mm from the nominal); hence, the diameter value used in calculating stress is measured as the average of the diameters measured at five equally spaced locations within each tested sample.

### 2.4. Mechanical Characterization of Specimens 3D Printed from the Reinforced Filaments 

The properties of 3D-printed components can differ from those of their constituting filaments, as 3D printing processes can introduce defects, voids, and different types of heterogeneities [1,37]. This motivated investigating the mechanical properties of parts 3D printed using the reinforced filaments fabricated in this work. To this end, the stress-strain curves of cylindrical samples 3D printed using the fabricated sand and SiC-reinforced PLA filaments are obtained. Cylindrical samples have a diameter of 10 mm and length of 10 mm and are printed from sand- and SiC-reinforced PLA filaments using a LulzBot Taz 6 3D printer. Samples are loaded in compression under displacement loading conditions. The same strain rate (i.e., 5.6 × 10^−4^ s^−1^) applied to the tensile-tested filament samples is applied to the 3D-printed cylinders. For each fabricated reinforced filament, at least three samples were printed and tested. Each 3D-printed cylinder requires approximately 60 cm of filament and a printing time of 45 min. This material and time cost motivated using a minimum of 3 samples per weight fraction. To compute the engineering stress and strain, the force and displacement reported by the machine’s load cell and internal actuator are normalized by the sample’s length and cross-sectional area, respectively. The cross-sectional area of a sample is calculated using the average diameter computed by averaging the diameters measured at four equally spaced locations along the sample.

## 3. Results and Discussion

### 3.1. Spatial Dispersion of the Reinforcing Particles from a Microscopy Perspective 

**Figure 7:**
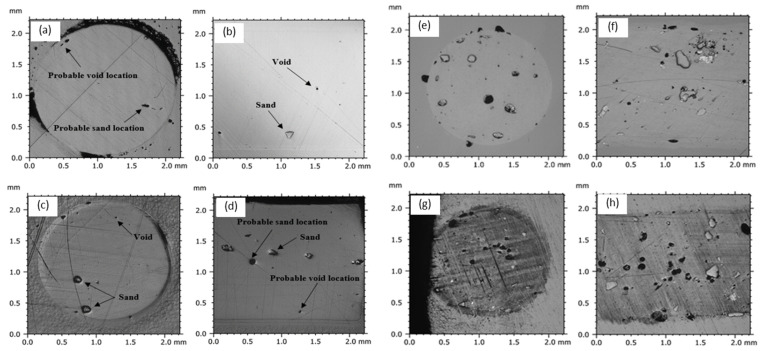
Microscopy images showing the dispersion of sand particles along the transverse and longitudinal filament directions. Images (**a**,**b**) represent the transverse and longitudinal directions of the 0.5 wt% sand reinforced filaments. Similarly, (**c**,**d**), (**e**,**f**), (**g**,**h**) correspond to the 1.0, 10, and 15 wt% sand, respectively. Horizontal lines are added to highlight the filament boundaries.

The samples of the fabricated filaments were polished using the methodology discussed in Section 2.2. Subsequently, microscopy was used to investigate the spatial distribution of the particles in the polished specimens. Figure 7 provides insights into the distribution of the reinforcing particles within the PLA-sand-fabricated filaments at low and high reinforcement weight fractions. The figure comprises microscopy images that show the particles’ distribution along the transverse (i.e., across filament diameter) and longitudinal (i.e., along filament axis) filament directions. Images show both particles and voids made by dislodged particles during polishing. Figure 7 demonstrates that particles are randomly dispersed across the transverse and longitudinal filament directions. Moreover, minimal clustering is observed. 

The observed random distribution of particles and minimal clustering indicates that the particles are relatively well-dispersed across the filaments. The latter qualitatively indicates that using particle-coated pellets with single-screw, short-nozzle low-cost extruders can substitute for the in-extruder mixing stage, which requires two-screw long-nozzle industrial extruders. Microscopic results qualitatively indicate that the low-cost fabrication process successfully produced filaments with well-dispersed particles, particularly along the filament’s longitudinal direction. The emphasis on the longitudinal direction is highlighted, as the longitudinal homogeneity of filaments is critical to FDM 3D printing processes. During 3D printing, layers of a filament are deposited in a sequential manner to build the part. This inherently makes the layering direction the weakest direction in 3D-printed components [38]. Any longitudinal heterogeneity (e.g., due to clustered particles) in used filaments can further undermine the properties along the layering direction. 

**Figure 8:**
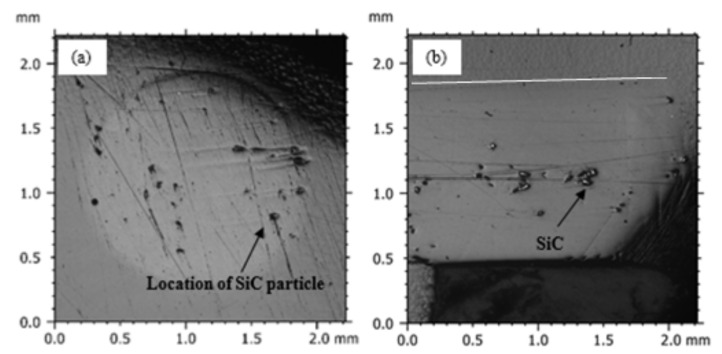
(**a**) Cross-sectional and (**b**) longitudinal view of 2 wt% PLA-SiC filament. Horizontal lines are added to highlight the filament boundaries.

The deductions made using the microscopic results of the sand-reinforced PLA filaments apply also to the SiC-reinforced PLA filaments. Figure 8 shows the SiC-particles distribution along the transverse and longitudinal direction of a 2 wt% SiC-reinforced filament. Filaments with a SiC weight fraction of 2 wt%, which is the highest reinforcement level used, are most suited to describe particle dispersion in the fabricated filaments. At lower weight fractions and since SiC particles are small, microscopy images might show a very small number of particles, which does not help in shedding light on particle distribution. Figure 8 shows randomly dispersed particles and minimal clustering, corroborating the microscopy results of the PLA-sand filaments. 

Microscopy images are useful in qualitatively evaluating the reinforcing particles’ distribution. However, microscopy images capture a few sections of the infinite sections comprising a filament. Thus, they provide local information. The microscopy results are complemented in the next section by stress-strain data describing the macroscopic behavior of the reinforced filaments. Combined, macroscopic behavior and microscopy results can better shed light on the effect of reinforced particles as well as their dispersion. 

### 3.2. Mechanical Behavior of Dune Sand and SiC Reinforced PLA Filaments 

**Figure 9:**
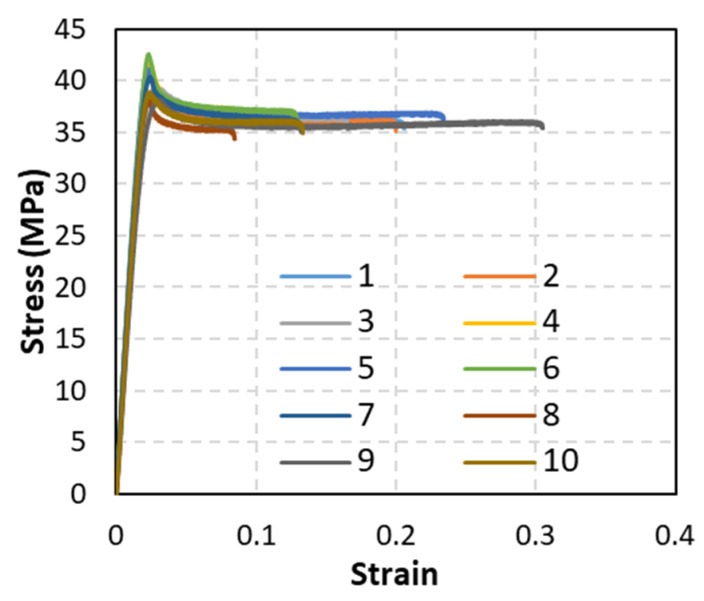
Stress-strain curves of PLA filaments (not reinforced).

**Figure 10:**
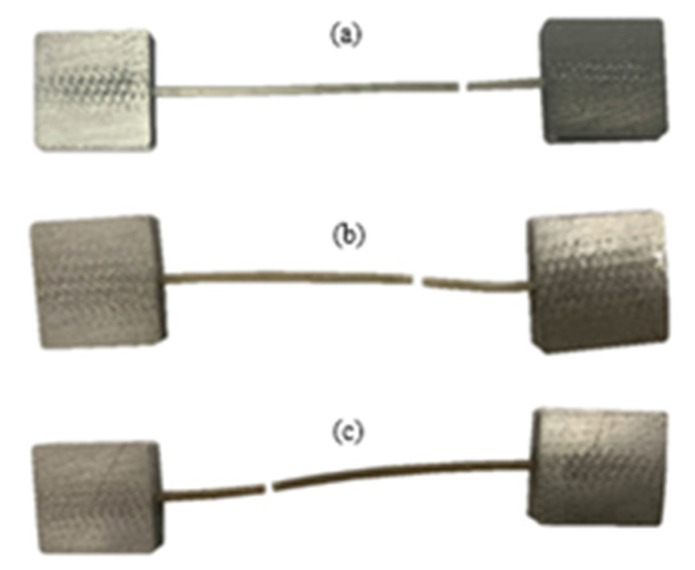
Images of uniaxially loaded PLA filaments. Representative samples (**a**–**c**) show that failure occurred at different locations but at a substantial distance from the loaded ends and confirm the absence of necking.

The behavior of pure PLA filaments was investigated first to ensure that the produced PLA filaments were consistent with commercially available PLA filaments and to establish a reference that facilitates observing the effects of reinforcing particles. To this end, 10 pure (without adding particles) PLA filament specimens were fabricated and tested under uniaxial loading following the testing protocol described in the methodology section. The tensile stress-strain curves obtained from the 10 pure filament specimens are shown in Figure 9. The behavior observed is very repetitive and representative of PLA filament behavior, which comprises three phases: elastic, yield followed by softening, and plateau stress that ends abruptly [38]. The stiffness, ultimate strength, and plateau stress of the 10 specimens exhibited marginal variations. On the other hand, the failure strain, which describes the macroscopic strain in the specimen’s gauge area at the onset of failure, showed significant variation, with failure strain values ranging between 0.08~0.3. This indicates that failure in the specimens occurred due to defect-triggered brittle failure. Images of the tested specimens, which are shown in Figure 10, confirm the absence of necking.

From the stress-strain curves shown in Figure 9, the average stiffness and tensile strength of the tested PLA filaments were calculated as 2.13 MPa and 40.02 GPa, respectively. Stiffness was calculated using the slope of the stress-strain curves in the elastic region, while strength was defined as the highest stress values realized by the stress-strain curves. The determined stiffness and strength values of fabricated PLA filaments agree with PLA properties reported in the literature [39,40].

**Figure 11:**
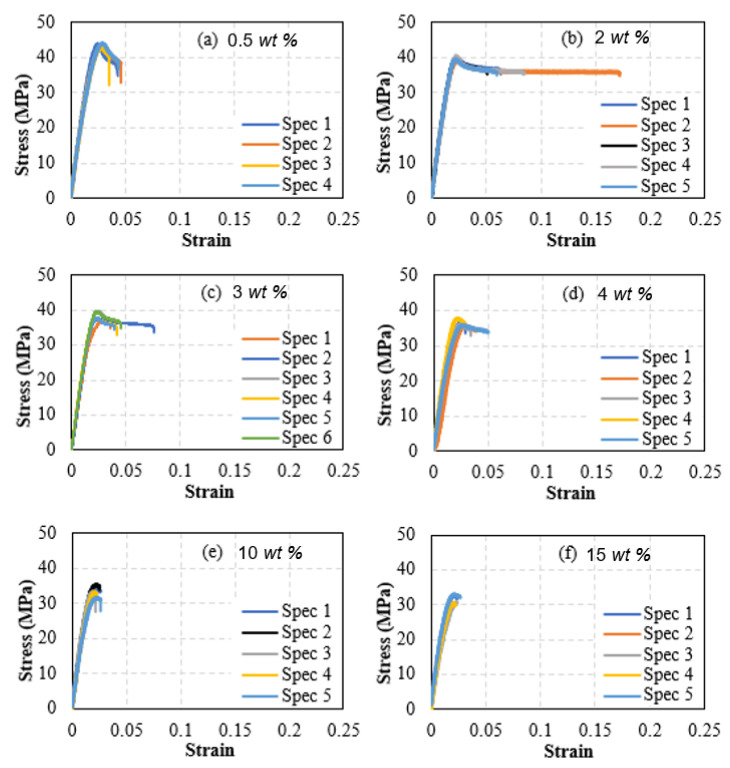
Stress-Strain curves of PLA filaments reinforced with fine dune sand. Subfigures (**a**–**f**) correspond to the sand weight fractions of 0.5%, 2%, 3% 4%, 10%, and 15%, respectively.

**Figure 12:**
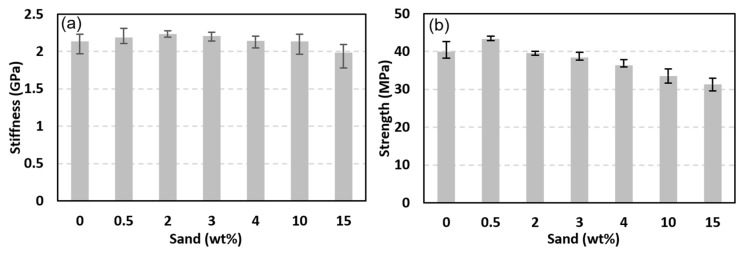
Effect of sand weight fraction on PLA-sand filaments’ stiffness (**a**) and strength (**b**).

The effect of including sand-reinforcing particles on the mechanical properties (stiffness and strength) of PLA filaments was measured by performing uniaxial tensile tests on sand-reinforced filaments. Five sand-reinforced filament specimens, in general, were tested at each of the sand fraction ratios 0.5, 2, 3, 4, 10, and 15 wt%. The stress-strain curves of the sand-reinforced filaments are shown in Figure 11. The stiffness and strength of the sand-reinforced filaments were calculated from Figure 11 and are reported in Figure 12. For comparison, the stiffness and strength of the pure PLA filaments are included in the figure. The spread and scatter in the determined stiffness and strength data for both stiffness and strength are presented using error bars in Figure 12. The error bars were defined as the difference between the maximum and mean of the measured stiffness and strength values. According to Figure 12, sand particles have a nonmonotonic effect on stiffness. Adding sand particles, up to 10 wt%, can increase the stiffness of sand reinforced PLA filaments. However, introducing more reinforcing sand particles, beyond 10 wt%, decreases PLA filaments stiffness significantly. For instance, adding 15 wt% sand particles reduced the PLA filaments’ stiffness by 6.8%. In the reinforcement range with a positive effect on stiffness (i.e., less than 10 wt%), the highest increase in stiffness was observed at the reinforcement levels of 2~3 wt%. At the latter reinforcement levels, the stiffness of the reinforced filaments was higher than that of the pure PLA filaments by 4.5%. Reinforcing PLA filaments with sand particles reduced its ductility. The reduction increased with increasing the reinforcement level. At the highest level of reinforcement, 15 wt%, an almost glassy behavior was observed, with failure occurring at the onset of maximum stress. Images of the tested specimens followed the behavior observed in Figure 10. The failure occurred at random locations away from the loading boundaries and the necking behavior was not present.

The effect of introducing sand particles on the strength of PLA filaments followed a nonmonotonic trend similar to that of stiffness. However, the highest strengthening effect was observed at a much lower sand particles content (0.5 wt%). The strength of the filaments with 0.5 wt% sand particles was higher than that of the pure PLA filaments by 8.3%. Including sand particles at ratios above 2~3 wt% decreased the strength of PLA filaments. For instance, the strength of the filaments with the largest sand content (i.e., 15 wt%) was lower than that of pure PLA by 21.7%. Results, particularly Figure 11, show that the tested filaments provided a consistent response. This indicates that reinforcing particles were well-dispersed in the filaments. Moreover, results of Figure 10 demonstrate that one can tune the stiffness and strength of PLA using dune sand particles. However, sand content should be less than 2 wt% in general to realize improved stiffness or strength values. In addition, optimizing PLA-sand filaments’ stiffness requires a different sand content than that needed to optimize their strength.

**Figure 13:**
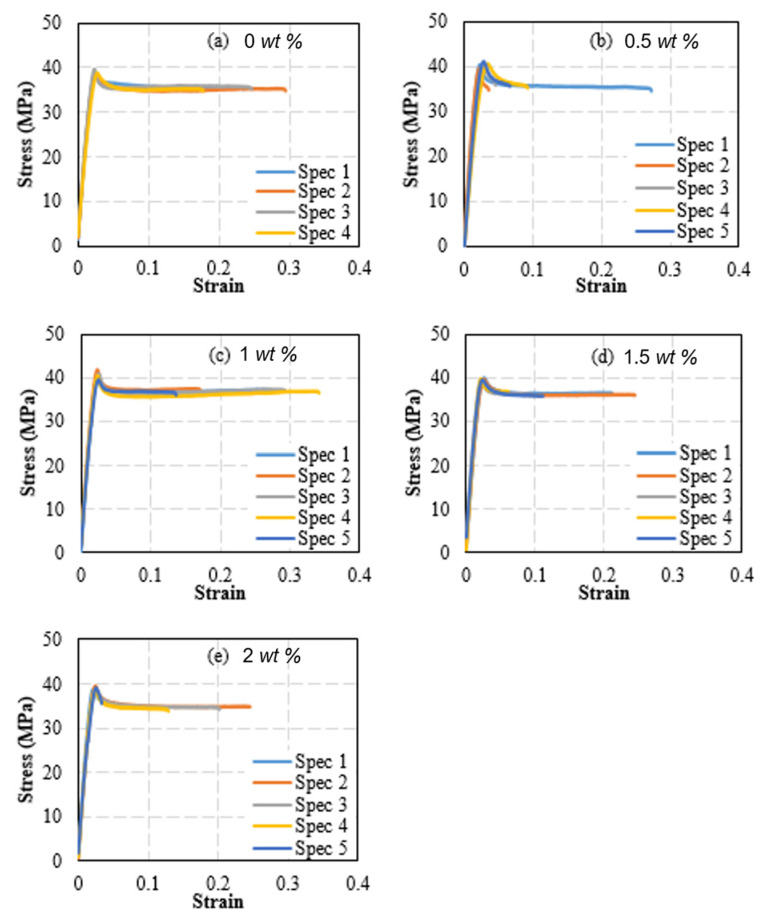
Stress-Strain curves of PLA filaments reinforced with silicon carbide. Subfigures (**a**–**e**) correspond to carbide weight fractions of 0%, 0.5%, 1%, 1.5%, and 2%, respectively.

**Figure 14:**
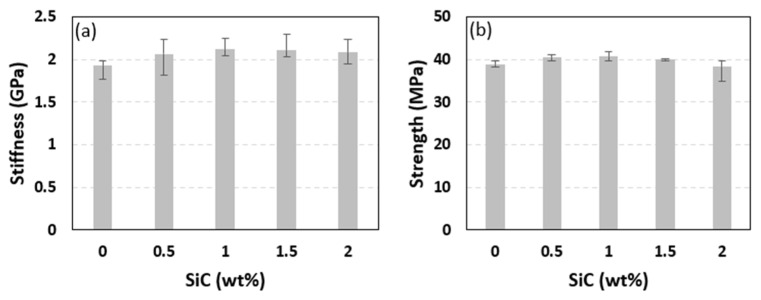
Effect of SiC weight fraction on PLA-SiC reinforced filaments’ stiffness (**a**) and tensile strength (**b**).

The effect of including SiC particles on the tensile properties of PLA filaments was measured through uniaxially testing PLA filaments reinforced with 0.5, 1, 1.5, and 2 wt% SiC. The PLA pellets used to produce the PLA-SiC filaments were from a different batch than the one used to fabricate the PLA-sand pellets. Accordingly, un-reinforced PLA filaments were fabricated and tested. These were used to calculate the stiffness and strength of the PLA filaments fabricated from the second batch. The stress-strain curves obtained from testing the un-reinforced filaments as well as the SiC-reinforced filaments are presented in Figure 13. Five specimens were tested at each reinforcement level. However, to characterize the un-reinforced PLA, four specimens were tested as the stress-strain behavior of the un-reinforced PLA filaments is not expected to exhibit significant scatter. Figure 13 shows repeatable stress-strain curves in terms of stiffness, strength, and softening at every reinforcement level. Failure strain varied significantly among samples with the same SiC content, indicating brittle failure. As for the case of un-reinforced PLA, images of tested specimens indicated that the failure occurred in a brittle manner, necking was not present, and the failure occurred at arbitrary locations away from the boundaries. The stiffness and strength of the SiC-reinforced filaments were calculated from the stress-strain curves of Figure 13 and are presented in Figure 14. The stiffness and strength of the unreinforced PLA filaments are included in the latter figures for comparison. Both the stiffness and strength followed trends that resembled those observed in sand-reinforced filaments. Stiffness variations with SiC particles content followed a nonmonotonic behavior. The stiffness increased with the introduction of 0.5 and 1 wt% SiC. At 1 wt%, the highest stiffness was observed, which represented a 10.1% increase, as compared to un-reinforced PLA. The stiffness plateaued between the SiC weight fractions of 1~1.5 wt%. However, the stiffness seemed to reach an infliction point at 1.5 wt% SiC, and commenced to exhibit a decreasing trend at SiC concentrations higher than 1.5 wt%. Yet, at 2 wt% SiC, the stiffness was 8.2% higher than that of un-reinforced PLA.

### 3.3. Effect of Reinforcements on the Mechanical Properties of 3D Printed Samples

**Figure 15:**
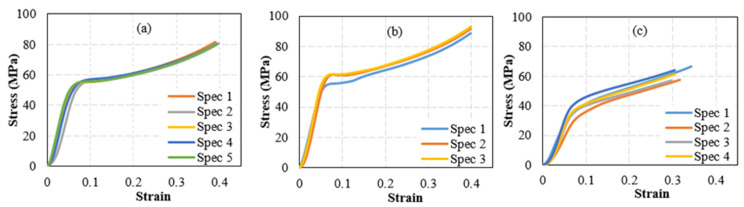
Stress-Strain curves of 3D printed PLA-sand compression cylindrical specimens. Subfigures (**a**–**c**) correspond to the sand weight fractions of 0, 0.5, and 15 wt%, respectively.

Filament deposition of 3D printing is known for introducing manufacturing-induced imperfections in fabricated parts [37]. Common imperfections include voids and residual stresses. In addition, the layer-by-layer deposition, depending on the deposition orientation, can result in heterogeneous properties, even when the parent filament used to build the 3D-printed part is homogenous and isotropic. As printing-induced defects can interact with the reinforcing particles, the effect of reinforcements on the properties of 3D-printed parts can be different than their effect on the properties of filaments. In addition, from a statistical perspective, the distribution of reinforcements in filaments and 3D-printed specimens can be dissimilar. The distribution of reinforcements in printed parts can be more uniform due to their larger size and print overlay. Accordingly, it is instrumental to assess the effect of reinforcing particles on the mechanical properties of 3D-printed parts. The effect of sand reinforcing particles on the mechanical behavior of 3D-printed parts is investigated by testing cylindrical specimens printed using PLA-sand filaments with 0.5 and 15 wt% sand. These weight fractions were selected as they produced the best and worst behaviors at the filament level. The stress-strain responses representing the compressive behavior of the tested cylindrical specimens are reported in Figure 15. Cylindrical specimens printed using un-reinforced PLA filaments were also tested for comparison. The stress-strain curves of the latter specimens are included in Figure 15.

The stress-strain curves of the un-reinforced PLA cylinders, Figure 15a, showed different behavior than that exhibited by the un-reinforced PLA filaments. Under compressive loading, significant hardening behavior was observed, whereas PLA filaments exhibited a softening phase that was followed by a plateau phase. The lack of a hardening phase in the case of PLA filaments is a typical behavior of thermoplastics under tensile loading. In addition, it can be, in part, related to their slender fiber-like geometry. The stiffness and yield strength of the PLA cylindrical filaments were computed as 1.17 GPa and 49.4 MPa. The yield was determined using the 0.2% offset approach. 

The stiffness of 3D-printed PLA cylinders is notably lower (less by more than 50%) than that of PLA filaments. On the other hand, the yield of the 3D printed cylinders is 23% higher than the strength exhibited by the PLA filaments. These results agree with the literature that show that the tensile stiffness of PLA can be higher than its compressive stiffness, and the tensile yield strength of PLA is lower than its compressive yield strength [41]. 

**Figure 16:**
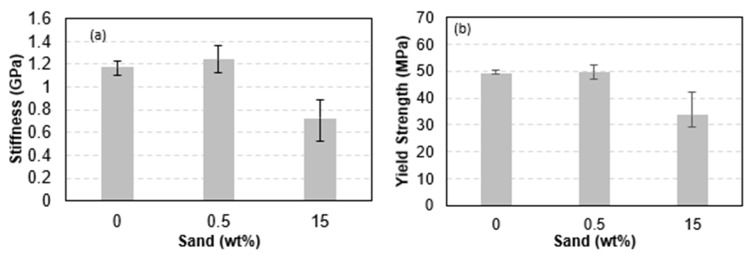
Properties of 3D printed PLA-sand compression cylindrical samples, showing (**a**) stiffness and (**b**) yield strength.

In terms of trends, introducing sand reinforcing particles to the 3D printed cylinders resulted in a similar effect to that observed in the case of reinforced filaments. Adding a small sand weight fraction resulted in a stiffening and strengthening effect, as seen in Figure 16, which was obtained from Figure 15. The stiffness of 1 wt% sand-reinforced cylinders increased by 6% as compared to un-reinforced PLA cylinders. On the other hand, introducing large sand weight ratios lessened the cylinders’ stiffness. The stiffness of 15 wt% sand-reinforced cylinders decreased by 38% as compared to un-reinforced PLA cylinders. Similarly, the strength of the reinforced 3D-printed filaments increased by introducing 1 wt% sand, namely increased by 11% as compared to the strength of the nonreinforced PLA cylinder. On the other hand, the strength of the reinforced 3D-printed cylinders decreased significantly at high sand content. At 15 wt% sand content, the strength decreased by 32.5%. While reinforcing particles, such as sand, can contribute positively to the matrix’s stiffness and strength by assisting in sharing the internal loads, increasing their content can result in a reversed effect [42]. Increasing the reinforcing particle content increases the probability of microstructural defects (e.g., lack of bonding at the particle-matrix interface) and decreases the uniformity of stress distribution in the matrix. The latter can cause, in multiple locations, localized stresses that exceed the yield of the polymer. These areas would exhibit softer stiffness; thus reducing the stiffness of the reinforced matrix. In addition, increasing stress heterogeneity in the presence of higher defects probability increases the potential for localized failure. The latter explains the reduced yield strength of the highly reinforced 3D-printed cylinders.

**Figure 17:**
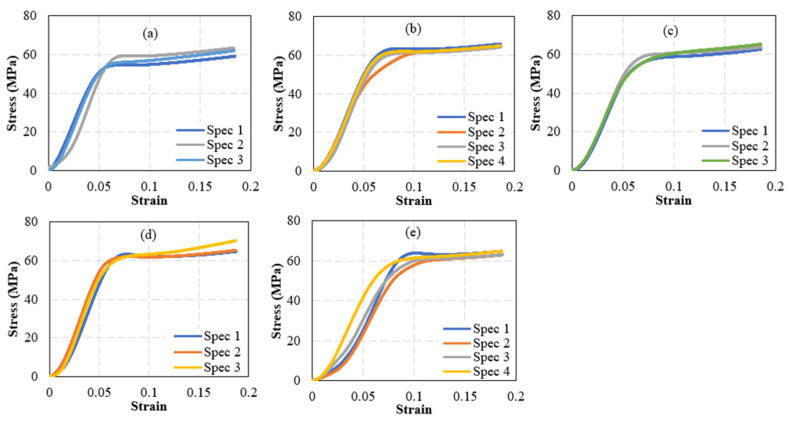
Stress-Strain curves of 3D printed PLA-SiC compression cylindrical specimens. Subfigures (**a**–**e**) correspond to the SiC weight fractions of 0, 0.5, 1, 1.5, and 2 wt%, respectively.

**Figure 18:**
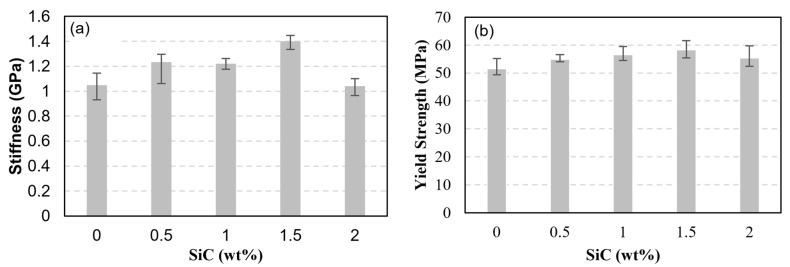
Properties of 3D printed PLA-SiC compression cylindrical samples, showing (**a**) stiffness and (**b**) yield strength.

The effect of SiC particles was assessed by testing cylinders 3D printed using PLA-SiC filaments. The stress-strain curves of the cylinders, which had SiC weight fractions of 0, 0.5, 1, 1.5, and 2 wt%, are shown in Figure 17. The stress-strain curves show significant hardening, as in the case of sand-reinforced PLA cylinders. Thus, in terms of hardening, both PLA-sand- and PLA-SiC-based 3D-printed cylinders showed different behavior than that of their filaments. To better quantify the effect of SiC particles, the stiffness and yield strength of the SiC-reinforced cylinders were calculated using Figure 17 and are shown in Figure 18. It is worth reminding that the batch of PLA used to print the SiC-reinforced cylinders is different from that used to print the sand-reinforced cylinders. According to Figure 18, small reinforcement content has stiffening and strengthening effects. At 1.5 wt% SiC, the stiffness of the reinforced cylinder was higher than that of its nonreinforced counterpart by 33%. On the other hand, at 1.5 wt% SiC, the yield strength of the reinforced cylinder was higher than that of the nonreinforced PLA cylinder by 13%. Increasing the SiC content beyond 1.5 wt% resulted in a decrease in the stiffness and strength of the reinforced 3D-printed cylinders.

At small reinforcement contents, both sand and SiC particles qualitatively affected the behavior of the 3D-reinforced cylinder in a similar manner. They both introduce a stiffening and strengthening effect. However, the particles differed in their stiffening and strengthening magnitudes. Nevertheless, the stiffening and strengthening effects observed were in the single digit range (less than 10%). Though such magnitudes are small, they can have a significant impact. An increase in the filament’s stiffness and strength can allow for using reduced in-fill during printing. Given that PLA is widely used, reduced in-fill can lead to significant material savings. This argument can apply to a wide range of filaments. The results showed that the proposed low-cost fabrication process can be used to tailor the stiffness and strength of thermoplastic filaments, which is one of the objectives of this work. Moreover, the results showed that both reinforced filaments and cylinders printed from them produced consistent behavior in terms of stress-strain behavior, stiffness, and strength. Consistency in behavior indicated that the reinforcing particles were well-dispersed in the filaments and printed cylinders. This suggests that the process used to produce the filaments was effective in dispersing the reinforcing particles.

## 4. Conclusions

This study proposed a low-cost process for fabricating reinforced-thermoplastic filaments. The process is geared toward the desktop FDM 3D printing community and utilizes tools readily available to the community, namely, low-cost desktop single-screw extruders. To overcome the inability of these extruders to readily mix different constituents and fabricate multiphase filaments, the proposed approach utilized multiphase pellets in the form of plastic pellets coated with the reinforcing material. The pellets were coated by manually mixing them with preheated reinforcing particles. The viability of the process was proved through two case studies, namely, by fabricating sand-reinforced and SiC-reinforced PLA filaments. Optical microscopy images showed that the particles were randomly dispersed, and minimal clustering was observed. Moreover, mechanical tests showed that the stress-strain, stiffness, and strength of the fabricated pellets were consistent and repeatable; thus indicating that the reinforcing particles were well-dispersed in the filaments.

Mechanical tests showed that improving the stiffness or strength of PLA filaments requires utilizing relatively small weight fractions of the reinforcing phase. In the case of sand-reinforced PLA filaments, best enhancements in stiffness (4.5%) and tensile strength (8%) were observed at the sand weight fractions of 2 wt% and 0.5 wt%, respectively. In the case of SiC-reinforced PLA filaments, the best enhancements in stiffness (9%) and yield strength (5%) were observed at the SiC weight fraction of 1 wt%.

The compatibility of the produced reinforced filaments with commercial desktop printers was assessed by printing cylindrical samples using PLA-sand and PLA-SiC filaments. The printed cylinders were tested under compressive loading. In the case of PLA-sand cylinders, the best enhancements in stiffness (5%) and strength (1.2%) were observed at the sand weight fraction of 0.5 wt%. On the other hand, for the PLA-SiC cylinders, the best enhancement in stiffness (33%) and yield strength (13%) were observed at the SiC weight fraction of 1.5 wt%.

## Data Availability

The data that support the findings of this study are available from the corresponding author upon reasonable request.
